# Tailoring the Interface of Biomaterials to Design Effective Scaffolds

**DOI:** 10.3390/jfb9030050

**Published:** 2018-08-21

**Authors:** Ludovica Parisi, Andrea Toffoli, Giulia Ghiacci, Guido M. Macaluso

**Affiliations:** 1 Centro Universitario di Odontoiatria, Università degli Studi di Parma, Via Gramsci 14, 43126 Parma, Italy; andrea.toffoli@unipr.it (A.T.); giulia.ghiacci@gmail.com (G.G.); guidomaria.macaluso@unipr.it (G.M.M.); 2 Dipartimento di Medicina e Chirurgia, Università degli Studi di Parma, Via Gramsci 14, 43126 Parma, Italy

**Keywords:** tissue engineering, biomaterials, chitosan

## Abstract

Tissue engineering (TE) is a multidisciplinary science, which including principles from material science, biology and medicine aims to develop biological substitutes to restore damaged tissues and organs. A major challenge in TE is the choice of suitable biomaterial to fabricate a scaffold that mimics native extracellular matrix guiding resident stem cells to regenerate the functional tissue. Ideally, the biomaterial should be tailored in order that the final scaffold would be (i) biodegradable to be gradually replaced by regenerating new tissue, (ii) mechanically similar to the tissue to regenerate, (iii) porous to allow cell growth as nutrient, oxygen and waste transport and (iv) bioactive to promote cell adhesion and differentiation. With this perspective, this review discusses the options and challenges facing biomaterial selection when a scaffold has to be designed. We highlight the possibilities in the final mold the materials should assume and the most effective techniques for its fabrication depending on the target tissue, including the alternatives to ameliorate its bioactivity. Furthermore, particular attention has been given to the influence that all these aspects have on resident cells considering the frontiers of materiobiology. In addition, a focus on chitosan as a versatile biomaterial for TE scaffold fabrication has been done, highlighting its latest advances in the literature on bone, skin, cartilage and cornea TE.

## 1. Introduction

Biomaterials are discernable from any other material for their ability to co-exist with biological tissues without causing adverse effects. In the last century, this unique property has led to a growing interest from scientists for the development of medical devices. However, since the manner in which biomaterials and tissues could interact are numerous, the concept of biocompatibility, often superficially defined as “biological inertia”, requires a deeper discussion on its significance.

To the first generation of biomaterial scientists, who developed and introduced the use of implantable medical devices (1940–1980), it was quite obvious how the optimal performance was obtained when no adverse effects were triggered in the host [1]. Consistently, the definition of biocompatibility soon became a list of negative issues that should be avoided, such as non-toxicity, non-immunogenicity, non-carcinogenicity, non-irritancy and so on. The evidence that: (i) the response of specific material varied from the site of its application; (ii) in an increasing number of clinical needs material should evoke appropriate responses of the surrounding tissues; and (iii) that sometimes materials should be degraded after having carried out their function; moved to a re-evaluation of the concept of biocompatibility, which was defined in 1987 as follows:

“Biocompatibility refers to the ability of a material to perform with an appropriate host response in a specific situation” [1].

However, this definition was still in its infancy and according to Williams it should be revised. Particularly, biocompatibility is accomplished when a stable equilibrium between the biomaterial and the host is obtained. As such, we have to consider that both the clinical intervention and the unique characteristics of each individual are of considerable significance when defining the concept [2]. Thus, the concept of biocompatibility should be adapted on the clinical need we have [3].

Within the set of long-term implantable medical devices, we challenge a wide spectrum of conditions ranging from hard to soft tissue. In this specific case, we should talk about clinical biocompatibility, which requires the use of mechanically stable non-degradable materials and functionally suitable to definitively support or replace the damaged organ. Examples of these applications may be titanium and its alloys for dental and orthopedic applications, silicones for ophthalmological devices and breast implants, or polytetrafluoroethylene (PTFE) for heart valves and vascular grafts reconstruction [4,5,6,7,8].

The concept of biocompatibility needs then to be further modified to include the set of degradable implantable materials; devices that have to perform a function for a restricted time frame. A clear example of this type of materials are synthetic polymers, i.e., that commonly used to produce suture materials. With this type of materials, degradability should be included in their biocompatibility definition [9,10,11].

The paradigm of biocompatibility could be still enhanced to include transient invasive intravascular/intraparenchymal devices, such as catheters for the delivery of substances for nutritional, diagnostic and therapeutic purposes. Clearly, the choice of material must relapse on that with good blood compatibility, in order to avoid unsought effects as thrombosis [12].

All the biomaterials applications mentioned above, as the relative concept of biocompatibility, have been largely studied and have well-known clinical success, but a fundamental shift in the mechanism of biocompatibility has to be done to approach the vast field of scaffold development for tissue engineering applications.

## 2. Tissue Engineering

In the last decades, a fundamental shift in the design and use of biomedical materials has been witnessed when the era of regenerative medicine (RM) began. RM is an interdisciplinary field that combines the principles of engineering and life sciences for the goal of building tissues and organs damaged by age, disease, trauma or congenital defects. All conditions that without intervention would spontaneously remain unrepaired because the body possesses only a limited ability to regenerate. RM encompasses different strategies, including pharmaceutical targeting of bioactive factors for in situ stimulation of stem cells, genetic engineering of autologous cells followed by their grafting or the combination of cells, soluble factors and biomaterials to promote tissue regeneration; this latter approach is known as tissue engineering (TE) [1,13].

**Figure 1:**
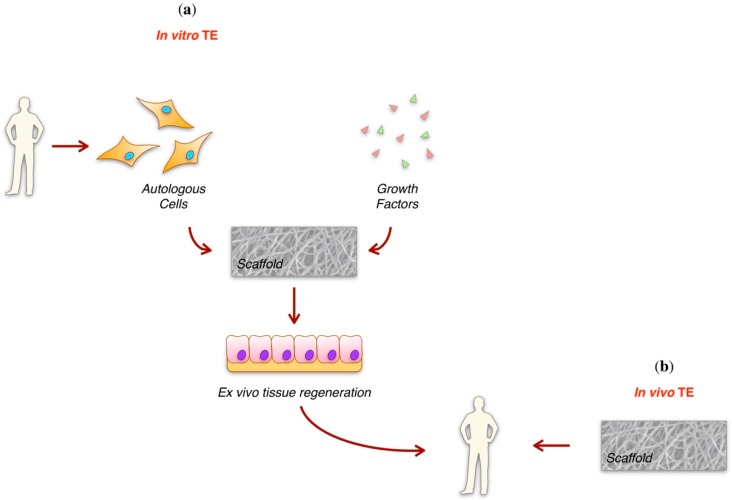
Tissue Engineering. (**a**) In vitro TE. Autologous cells and growth factors are co-seeded on the biomaterial scaffold and maintain in culture until tissue neo-formation. Tissue regeneration occurs ex vivo and once formed, the tissue is grafted; (**b**) In vivo TE. Biomaterial scaffold is directly implanted in the damaged anatomical site. Tissue regeneration occurs in vivo.

In particular, TE paradigm may follow an in vivo or an in vitro approach (Figure 1), but it always relies on the use of a biomaterial, which has to be molded in a 3D scaffold. The scaffold acts as a template to accommodate cells, which adhere, proliferate, differentiate and start to depose new extracellular matrix (ECM), which progressively coat the structure, leading to the regeneration of the new tissue, while the biomaterial is progressively resorbed [1].

In such a complex scenery, it becomes obvious that the concept of biocompatibility needs to be dramatically revised, since the scaffold should be thought as a high dynamic structure able to elicit different responses, in different tissues or organs. The goal is the induction of regeneration where the naturally occurring reparation is scar, with consequent impairment of tissue/organ function. In this arena, the scaffold should encompass different features: it should determine the shape of the defect and promote an appropriate cell response through the control of their phenotype and gene expression profile. Not less important, great significance is assumed by scaffold biodegradability.

## 3. Scaffold Requirements

Simple observation of biological tissues shows that they are extremely complex systems and that their structural organization relies on their functions [13]. The first step towards scaffold fabrication is to identify a biomaterial that could be molded in order to match specific tissue properties allowing the creation of a milieu suitable for cell growth, proliferation, differentiation and ECM deposition for a successful regeneration. Criteria for the choice of a suitable biomaterial include the possibility (i) to be degraded without any systemic adverse effects on the host; (ii) to assume proper mechanical properties to mirror that of the host tissue; (iii) to assume a suitable internal architecture; and (iv) to elicit surrounding tissues biological response [14].

### 3.1. Biodegradability Requirements

TE scaffolds are not considered as permanent implants. Therefore, they should be reabsorbed with an opportune rate of degradation and colonizing cells to regenerate the tissue. Furthermore, if the scaffold does not degrade, undesired effects may occur, such as internal pore obstruction by in vivo events with consequent tissue necrosis [15]. The choice of a biodegradable biomaterial is thus of great importance with regard to TE and must be accurately done since both the bulk material and its degradation products have not to be inflammatory, mutagens, toxic, carcinogens and so on. For example, materials that may be used in small volumes, with short degradation sutures, cannot be adequate for the realization of complex scaffolds, because they may trigger adverse effects in the host [3].

Biodegradability, otherwise bioresorbability, consists of the capacity of a material to decompose over time as a result of biological processes, i.e., hydrolysis or enzymatic activity, and is often a peculiar property of a wide range of polymers. Biodegradable polymers may be of synthetic or natural origin. Here, we propose a brief review of those commonly used in TE [16].

#### 3.1.1. Synthetic Biodegradable Polymers

Synthetic polymers represent the largest group of biodegradable polymers. They are widely used in biomedical applications, since their properties (e.g., biodegradability, mechanical aspects, porosity) can be tailored for the specific application during their synthesis and many are also suitable for printing [17,18].

Among the synthetic polymers, aliphatic polyesters (-O-CHR-CO-)_n_-) are the most attractive group, since their esteric group is easily accessible by human enzymes. Their degradation rate can be controlled by monitoring their synthesis and polymerization conditions or introducing specific functional groups. Furthermore, their degradation products are recognized by the human body and can be removed by common metabolic pathways [16].

The most common alpha polyesters are polyglycolic and polylactic acids (PGA and PLA), which can be combined to form their copolymers. Both PGA and PLA have been shown to possess great capacity to support the attachment and proliferation of cells, especially those of bone and chondrocytes. Furthermore, the products of their degradation are glycolic and lactic acid, which are non-toxic and easily removable from the body by physiological metabolic pathways [17,19]. Poly-ε-caprolactone (PCL) is a viable alternative to the use of PGA/PLA polymers. PCL was one of the first synthetic polymers synthetized. Even though it has been forgotten for a long time because of its poor mechanical properties, it has been recently rediscovered thanks to its superior viscoelasticity and to its malleable rheological properties that allow its manufacturing into large-scale scaffolds [20].

#### 3.1.2. Natural Biodegradable Polymers

Natural polymers that include proteins or polysaccharides could be preferred to synthetic ones both because their repeating units are more similar to those commonly metabolized by the human body (amino acids and monosaccharides), and because they possess appropriate binding motifs for cells within their molecular structure [3].

Collagen, which is the most abundant structural protein of the human body, is probably the most used biopolymer in medicine. It is normally derived from animals (porcine, equine or bovine) and it has shown potential in bone and cartilage TE. Collagen possesses degradability properties: its biodegradation could be accomplished by natural lysosomal enzymes and collagenase, and most important it may be modulated by controlling its cross-linking during fabrication [21,22,23]. Another protein, which has recently shown great applicability in RM is silk fibroin, which is isolated from the cocoon of *Bombyx mori* silkworm and is easily degraded by human hydrolases [24,25].

On the other hand, polysaccharides are gaining interest among biomaterial scientists because of their capacity to trigger specific cell signaling [3]. Hyaluronan is one of ECM’s main component and thus it presents excellent biocompatibility with cells and tissues. Furthermore, hyaluronan possess excellent solubility in water, which contributes to a short residence time after its implantation and rapid resorption [26,27].

Alginate is a polysaccharide isolated from vegetal organisms (brown algae). It contains inflammatory components, but its purification contributes to limit this issue making alginate a suitable material for TE scaffolds, which do not elicit any host response within 1 year [28].

Chitosan which is derived from N-deacetylation of chitin, one of the main components of crustacean exoskeleton, is often used in scaffold manufacturing, and will be discussed later in this work.

### 3.2. Mechanical Requirements

Mechanical properties resembling those of the native tissue are among the first requirements an engineered scaffold should have. To be considered mechanically biocompatible, a scaffold should maintain the integrity of the defect until complete regeneration of the target tissue, meanwhile opportunely responding to external forces. At the same time, it has to possess fatigue properties to avoid its failure when undergoing cyclic loading.

Rheological parameters for proper scaffold design include (i) elastic modulus, that measures strain in response to a given tensile or compressive stress along the plane of the applied force; (ii) flexural modulus, that measures the relationship between a bending stress and the resulting strain after a compressive stress applied perpendicularly; (iii) tensile strength, that is the maximum stress a material can withstand before its break and (iv) maximum strain, that is the ductility exhibited by the material before a fracture.

**Figure 2:**
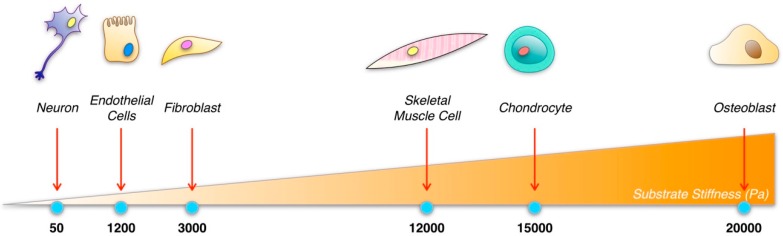
Cell phenotype is shaped by the stiffness of the substrate.

These properties, with particular regards to elastic modulus, in turn affect interstitial fluid flow, including nutrient and waste transport, which are of great importance for cell metabolism [29,30,31,32]. As their counterparts, tissue cells sense via mechanotransduction the stiffness and the mechanics of the surrounding milieu, that in human body range from hundreds of Pa (skin/subcutaneous tissue—57 Pa [33]) to GPa (trabecular bone—100 GPa [34]), to regulate their growth (adhesion, migration and spreading), proliferation and differentiation [35]. According to mechanobiology theories, cells cope well with the adhesion to substrates with stiffness similar to the tissue they belong to, as their way of migration along the material depends on its stiffness [36]. If the rigidity of the substrate is not in compliance with that of the native tissue, cells may switch their way of migration. This often occurs in pathological conditions, e.g., cancer metastasis, where physical properties of the tissue change and cells switch their way of migration from lamellipodia/filopodia to amoeboid mode [37]. In Figure 2, the substrates stiffness related to different cell phenotypes are reported.

The modes of cell adhesion and migration are of pivotal importance for cell differentiation and proliferation within the scaffold. Both these parameters have to be set also considering the function of the cells within the tissue they have to regenerate. Eventually, cell–material interactions, including adhesion, migration and spreading, define cell morphology, which is a key factor in triggering progenitor cells commitment [38,39]. It has been extensively shown that stiff substrates (30–35 kPa) promote osteogenic differentiation of mesenchymal stem cells, while softer ones (~10 kPa) allow myogenic or adipogenic commitment [40].

The options for TE include a wide range of materials, whose bulk mechanical properties contribute to the definition of their applicability in different applications. For instance, polysaccharides possess low strength and rigidity, that limit their use in soft tissue regeneration, while fibrous proteins, i.e., collagen, which normally provide stability and mechanical integrity to biological structures, may be exploited for hard tissues. Additionally, it has to be considered that the final mechanical properties of the scaffolds strongly depend on the manufacturing process, that often alters the mechanics of the bulk material. Considering this aspect, TE scaffolds of the very same material may be molded into porous, hydrogel and fibrous scaffolds.

#### 3.2.1. Porous Scaffolds

The first type of TE scaffolds that have been developed were 3D porous scaffolds, such as sponges and foams. Sponges and foams present a highly porous network, which is induced and controlled during the manufacture process. A limit for their application is often the low mechanical properties of the resulting substrates [17].

Event though, to the best of our knowledge, the literature on this topic is still lacking, a recent work by Li et al. investigated a fish collagen-based sponge as Dura mater substitute after surgical resection in rabbits. Animal were monitored during the postoperative period. All the animals recovered well and their wound healed properly. Histological analysis revealed that peripheral cells migrated into the outer layer of the implant, but no infiltration of brain cells was observed in the inner layer, indicating a good tolerance of the implant. Furthermore, neovascularization was observed after 30 days [41].

#### 3.2.2. Hydrogels

Hydrogels are networks of cross-linked polymers capable to swell with water. As such, they possess a biochemical similarity with highly hydrated glycosaminoglycan (GAG) organizing ECMs. This aspect has dressed hydrogels to an emerging part in the field of TE [42]. Methods for hydrogel fabrication are generally based either on chemical gelation, which include the formation of strong covalent bonds between the polymeric chains by secondary molecules interposition (chemical crosslinking), or on physical gelation, as a result of non-covalent interactions among the functional groups of polymer chains (physical crosslinking) [43,44]. It is clear that in the case of a chemical gelation, it is more than ever important to control the cytocompatibility of the chemistry exploited to design the scaffold, in order to avoid the introduction of potentially toxic components.

Moreover, recent advances in nano-fabrication techniques achieved the creation of hydrogels with a highly controlled structure. Regarding hydrogels mechanical characteristics, it is easily understood that they are materials with limited application in regenerative procedures of load-bearing tissues. However, these challenge may be at leas partly addressed by incorporating various materials, even in the form of nanoparticles, i.e., calcium phosphates or silica nanoparticles, to modulate their stiffness [45,46,47,48]. On the other hand, thank to their soft mechanical properties, polymeric hydrogels have been exploited for cell encapsulation and delivery, collecting great success especially for cardiac TE [49,50].

Considering hydrogels limited capacity of diffusion, they have been widely employed as materials for cartilage TE. For example, Zhang et al. prepared a copolymer hydrogel of PLGA and PEG by physical thermal crosslinking and loaded it with bone marrow mesenchymal stem cells for full-thickness articular cartilage defect repair. Specimens were in vivo tested in rabbits, which 12 weeks post-surgery showed enhanced fibrosis in control group (no treatment), partial restoration of the defect in gel group and great regeneration in gel/cells group [51].

Another suitable site for injectable hydrogel application is neural tissue. Chedly et al. prepared a chitosan-based hydrogel for spinal cord injury recovering in rats. They observed a reduction in astrocytes activation, which is typical during glial scar formation, and robust axon regrowth already 4 weeks after the the injury. Furthermore, the activation of inflammatory macrophages (M1) was up-regulated in the presence of chitosan 4 days after surgery, progressively decreasing in time until reaching maximum down-regulation with significant differences from control at 8 weeks of healing. According to this, reparative macrophages (M2) were statistically up-regulated in the presence of chitosan from 4 to 8 weeks [52].

#### 3.2.3. Fibrous Scaffolds

Developing structures to mimic tissue anatomy at a microscopic level is one of the major challenges in TE. The introduction of nanofibers has enhanced the chance to produce scaffold that potentially overcome this issue. Furthermore, nanofibers possess the advantage to supply a high surface/volume ratio, which, combined to their micro-porous structure, promotes peculiar cell responses. Methods to synthetize nanofibers include electrospinning, self-assembly and phase separation (see Section 3.3.1) [53].

Nanofibrous materials are probably the most explored scaffolds for TE and their application range from load-bearing hard tissues, i.e., bone or tendon/ligament, to vascular TE. However, their stiffness slightly limits their applicability to soft tissues.

Through the use of an electrospun nanofiber scaffold of polyhydroxybutyrate, chitosan and bioglass, Khoroushi et al. cultured and promoted the differentiation of human exfoliated deciduous teeth pulp cells into odontoblasts [54]. In another study, Chen et al. designed an electrospun gelatin/PLA scaffold blended with a hyaluronic-acid based hydrogel for articular cartilage repair showing complete tissue regeneration in rabbits 12 weeks after surgeries [55]. For tendon TE, Pauly et al. studied the effects of orientation and geometry of PCL electrospun nanofibers on scaffold mechanical properties by measuring rheological parameters, including elastic modulus, yield stress and strain. Interestingly, oriented nanofibers ameliorate the mechanical properties of the structured scaffold and at the same time boost elongation of adult human adipose-derived stem cells after 7 days of culture [56]. Interesting results on the application of nanofibers as scaffolds for TE come also from skeletal muscle and skin TE. Laminin-coated poly(methyl methacrylate) nanofibers showed to promote primary myoblasts proliferation, while nanofiber silk fibroin scaffold revealed to have great potential in wounds healing of skin in vivo [57,58].

### 3.3. Porosity Requirements

Another key characteristic that has to be considered during TE scaffold design is mass transport. Mass transport includes the carrier of nutrients, oxygen and waste to and by cells, as of molecules and signals [59]. In vivo these events occur by diffusivity and permeability through blood vessels, while in 3D models for TE mass transport could be obtained adjusting matrix porosity, which in the first step of tissue regeneration leads to the Brownian motion or fluid flow under applied pressure of substances, while hand to hand tissue is regenerating it should lead space and way for blood vessels and capillaries growth [60].

Furthermore, at a cellular level, porosity is of pivotal importance to allow cell migration and scaffold colonization. As such, it is obvious that pore size should be large enough to supply mass transport and not too large to prevent cell migration [61]. Pore size may range from few nanometers to millimeters and it has been shown how different pore sizes may influence different cell processes [62,63]. Nanometric dimensions of pore (d < 100 nm) promote cell attachment and functioning, while micrometric pores (100 nm–100 µm) allow the interaction and communication between cells through the extrusion of cytoplasmic protuberances [64]. Finally, the threshold pore size which allows cell migration across the structure depends on cell type and tissue function, i.e., hepatocytes which have a mean diameter of 20–40 µm preferred pore diameter of 20 µm, while bone cells (20–30 µm) according to the requirements of bone tissue need a pore diameter between 100 and 350 µm [65,66,67].

Scaffold porosity, pore size, geometry, distribution and interconnectivity, which in turn affect the mechanical properties, may be controlled by fabrication techniques, which can be divided in “top-down” approaches when the scaffold is pre-fabricated and in “bottom-up” methods, which consist in fabricating microscale tissue building blocks and assembling them to forme a larger construct.

#### 3.3.1. Top-Down Approaches

Traditional TE methods use “top-down” approach for scaffold construction, which consist in seeding cells onto a pre-fabricated scaffold. Generally, this type of approach has been engineered for thin and simple tissues, such as skin, cartilage or bladder [68,69,70]. Indeed, even though “top-down” methods allow to partially control scaffold porosity, they often present limitations to provide opportune diffusivity. Thus, considering that cells distance from nutrients can not exceed 200 µm, “top-down” approaches often present a limit for the regeneration of high density and high metabolic demanding organs and tissues, such as liver or kidney [71]. In spite of this, this type of approach is widely and successfully used in TE.

Solvent casting is the simplest, easy and inexpensive technique for scaffold preparation and it is totally based on solvent evaporation for the creation of a polymeric membrane (film). Since the main issue of this method is the impossibility to obtain pore formation, films obtained in this way have often been used in association with other porous scaffolds, as barriers [72]. To this purpose, Zonari et al. designed a bilayer skin TE construct combining a solvent casting poly(hydroxybutyrate-*co*-hydroxyvalerate) (PHBV) membrane for keratinocytes culture placed on the top of a 3D porous PHBV scaffold seeded with dermal fibroblasts [73].

To control the porosity, particularly pore size and geometry, porogens have to be used in “top-down” approaches. Porogens are components, such as salt, wax or sugars that blended in polymer solution are use to create pores and channels in 3D structures. Leaching techniques exploit a mold filled with porogens, in which polymer solution is cast and polymerized for solvent evaporation or crosslinking. After polymerization, porogens are thus leached away from the scaffold leaving a structure with the 94–95% of percentage porosity [74]. Porogen choice allow to control the size and the geometry of pores, but the design of a structure with accurate pore interconnectivity is still demanding [75]. Exploiting a combination of compression molding, heating, polymer etching and particle leaching, Baheiraei et al. developed a PCL-based scaffold with open pores up to 150 µm diameter for cardiac TE, which possess the capacity to support the cultivation of primary cardiomyocytes isolated from newborn rats [76]. A high porous 3D scaffold may be alternatively obtained through gas foaming technique, which consists in obtain pores by leaching porogens through high pressure gases [77]. Porous gelatin scaffolds were prepared by using hydrogen carbonate as a foaming agent by Poursamar et al., which were able to control pore diameter (280–550 µm) by the modulation of glutaraldehyde concentration as chemical crosslinking agent [78]. A recent advance in leaching techniques provide the use of soluble polymeric microspheres as an option to the use of porogens (melt molding), which can be used to avoid the use of potentially toxic organic solvents during scaffolds preparation [79].

The main challenge of the above cited techniques is the scarce possibility of controlling interconnectivity between formed pores. The introduction of controlled fiber deposition and freeze drying methods have greatly boost the research on the design of a scaffold with interconnected pores.

Freeze drying is based upon the principle of solvent (normally water) sublimation, which being a fast occurring transition leads to the formation of porosity and interconnectivity: as faster is the freezing as smaller are the generated pores [80]. Freeze drying may be applied both to produce 3D rigid structures either to preserve hydrogels structure until their hydration [73].

However, the most promising technique to produce porose and interconnected woven or non-woven scaffold is the electrospinning. Electrospinning is a commonly used method that exploits high voltage source to create an electrically charged polymer filament that forms fibers after drying, gelation or solidification. Electrospinning allows to have control both over fibers orientation and over pore geometry, consequently leading to the definition of interconnectivity. In the electrospinning process fibers diameter range from 50 to 1000 nm [81]. In the literature, most of the examples on nanofibrous electrospun scaffolds come from skin TE [82].

#### 3.3.2. Bottom-Up Approaches

All the methods cited among the “top-down” approaches do not allow to opportunely control scaffold geometrical cues, including internal micro-porosity and external macro-anatomy. As such, the emerging “bottom-up” techniques may hold great potential to address these issues, by fabricating tissue microscopic units, better known as building blocks, and combing them through multiple approaches in order to obtain a macroscopic complex organ or tissue [71].

Self-assembling constructs may be considered as the first generation of scaffolds designed through a “bottom-up” approach. Self-assembly method is based on the spontaneous organization of molecules into well defined and ordered structures, which clearly requires a deep understanding of building blocks and of their assembling dynamics and features [83]. By the adjustment of sample parameters, i.e., pH or temperature, it is possible to control self-assembly process and thus scaffold porosity and interconnectivity.

Even though self-assembly technique is still in its infancy, pioneering efforts for its use in TE have been already proposed in the literature. These initial efforts have been plagued by poor mechanical properties, difficulties in precisely governing 3D-morphology and high cost of production [84,85].

On the contrary, in the last decade addictive manufacturing (AM) techniques, otherwise known as rapid prototyping (RP) or three-dimensional (3D) printing approaches, have gained more and more attention because of the possibility of automation, good accuracy and reproducibility. The main appeal of these methods is the possibility to faithfully reproduce complex geometries of tissues designed with computer-aided design (CAD) systems, starting from 3D data acquired through imaging devices used for diagnosis, i.e., computer tomography (CT) or magnetic resonance imaging (MRI). This gives the concrete possibility to reach a patient-specific and customized therapy [86].

Basically, RP is based on the controlled deposition of a material in two-dimensional (2D) layers, whose sequential addition leads to the creation of a 3D object [87,88]. Among AM techniques, TE scaffolds may be ideally produced through free form fabrication (FFF), vat photopolymerization, which include stereolithography (SLA) and digital light processing (DLP), selective laser sintering (SLS) and inkjet three-dimensional (3D) printing.

FFF methods are the most commonly used for biocompatible scaffolds realization. In this case, 3D objects are created by guiding the extrusion of a polymeric fiber from a plotter on XYZ axis, which once deposited polymerizes by physical crosslinking, most often by cooling [89,90]. Bettahalli et al. demonstrated the possibility to monitor nutrient and oxygen perfusion and to supply cell viability of C2C12 myoblasts in vitro by using a FFF polymeric scaffold with controlled porosity. The main advantage of using FFF is the possibility to avoid the use of organic solvents, even though a remarkable limit includes the low level of resolution due to the dimensions of the extruded filament [90,91]. Alternatively, vat polymerization greatly enhances printing resolution offering an unprecedented possibility to control scaffold internal and external geometry by using a liquid photosensitive monomer resin that polymerize when expose to a light power source, most frequently an UV-source [90,92]. Vat polymerization methods include SLA and DLP: both have been used successfully, presenting significant advantages especially in fabricating vascular networks to promote in vivo angiogenesis, but cytotoxicity coming from the use of organic solvent and of uncured photo-initiators represents a major problem of these techniques [93,94,95].

SLS is another RP method with good resolution that uses a laser as power source to sinter 3D structures from several materials, including ceramics, metals and polymers [90]. Through SLS, in a ground-breaking experiment, Yingying et al. designed a PCL-hydroxyapatite scaffold with graded composition for osteochondral cartilage injuries. This model was tested in a rabbit model, which showed a complete cartilage-like repair 6 months after healing [96].

Finally, a smart alternative to the above described AM techniques is the 3D inkjet bio-printing, which relies on the use of cytocompatible ink in which cells may be encapsulated and directly printed as building blocks. The challenge in this case is the research of a suitable inkjet, which in primis should be compatible and capable to allow nutrients, oxygen and waste perfusion to cells [90]. Alginate, silk, collagen, gelatin and fibrin are currently the most suitable options. This point represent nowadays one of the most hot-topic in TE scaffolds design [97,98].

### 3.4. Bioactivity Requirements

According to Williams, this is the concept of biocompatibility in TE:

“The biocompatibility of a scaffold or matrix for a tissue engineering product refers to the ability to perform as a substrate that will support the appropriate cellular activity” [3].

As such, the concept of bioactivity, that is the capability of the material to establish a dynamic dialogue with its biological surrounding, is intrinsic. In other words, biocompatibility means that within its structure the scaffold should possess opportune stimuli recognizable by relevant cells for scaffold colonization and thus proper regeneration [3].

Scaffold biological activity may be promoted by enriching the scaffold with cues for cell adhesion, spreading, migration, proliferation and differentiation. Regarding cell adhesion and spreading, scaffold based on natural polymers have to be preferred to the synthetic ones, because biopolymers already present protein-related binding motifs suitable for cell adhesion [23,25,99,100,101]. However, many efforts have been done to confer docking points for cells also to synthetic materials, often by ameliorating protein adsorption at the interface [102,103,104]. Compositional gradients of inductive molecules may be created as driving force to promote cell migration, as well as combined to proteolytic components to create dynamic pathways for cell motility [35]. For example, bone morphogenetic proteins (BMP-2) or calcium ions may be used in bone TE to recruit bone cells, while abundances of laminin could be associated with angiogenesis and cancer invasion [105,106]. Furthermore, various kinds of ECM molecules have been demonstrated to have functions in regulating cell proliferation and differentiation. Thus, these molecules can be introduced onto 3D scaffolds to guide tissue commitment. For example, it has been observed that scaffold enrichment with polylysine inhibit neuronal differentiation to promote the glial one, while polyaniline and polypyrrole seem to have a role in osteogenic maturation [107,108].

Therefore, a key role in cell response to scaffold seems played by proteins, which are mostly non-specifically adsorbed on biomaterials surface shortly after their implantation. Therefore, the control of the amount, the composition and the conformation of adsorbed proteins may be a viable approach to design highly specific platforms for TE.

Two major aspects have to be considered in this sense: (i) the introduction of cues to elicit scaffold colonization and (ii) surface enrichment with molecular signals that specifically trigger cell fate and function.

#### 3.4.1. Control of Cell Adhesion

Cell adhesion on surfaces is mediated by ECM components, i.e., fibronectin, collagens and laminins, which possess binding motifs for the recognition of cell integrins. Integrins are a large family of homologous transmembrane receptors, constituted of two non covalently associated glycoprotein subunits, alpha (α) and beta (β), which after ligand binding, control cytoskeleton organization and thus cells grip to the matrix. In particular, after ECM recognition, the cytoplasmatic tail of the β subunit binds intracellular proteins that form mature focal adhesions, including talin, vinculin, α-actinin and filamin, and which in turn interacts with actin bundles to control cytoskeleton organization and cell adhesion, shape and migration [109].

Considering this essential point, the introduction of ECM-derived molecules to coat biomaterial surfaces may be an effective approach to ameliorate bioactivity requirements of TE scaffold. Historically, the use of entire ECM-derived proteins, particularly of fibronectin, has been considered the gold standard to coat and improve biomaterial surfaces, demonstrating enhanced cell adhesion and proliferation. However, to dispose of them in large quantities, these molecules are often isolated from other organisms and may elicit host immune responses [110,111,112]. More recently, the use of in vitro synthetized, decellularized and solubilized autologous ECM have shown great promises as coating, even though the set of opportune protocols for ECM dissociation avoiding the disruption of proteins assembled in the matrix structure is often demanding [113,114]. In addition, both for single molecules or for entire ECMs, their adsorption on surface may be influenced by material physic-chemical properties, as well as they are highly susceptible to in vivo enzymatic degradation. Therefore, the isolation of small peptides containing the cell binding domains may be a viable alternative. Cell binding domains are small amino acidic sequences that mediate cell attachment and that have been found in numerous ECM proteins [115,116]. The most popular is the Arginine-Glycine-Aspartic Acid (RGD) domain, highly conserved among the species from Drosophila melanogaster to humans and discovered by Ruoslahti and Pierschbacher in the early 1980s as the minimal recognition sequence within fibronectin [117].

**Figure 3:**
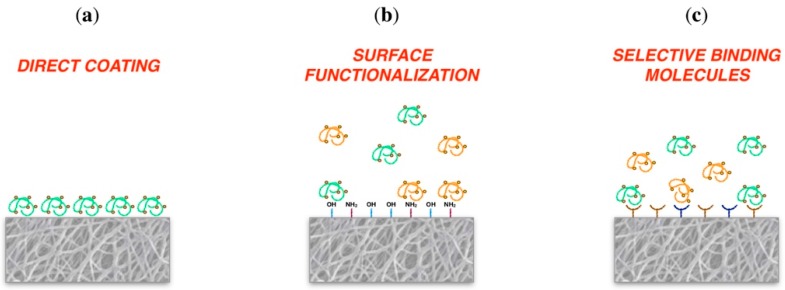
Control of cell adhesion through biomaterial coating with ECM-derived molecules. (**a**) Scaffold can be directly coated with the ECM-derived molecules; (**b**) Scaffold surface may be activated in order to expose functionalities able to bind ECM circulating molecules (i.e., fibronectin); (**c**) Selective binding molecules may be grafted on scaffold surface to retain ECM circulating.

Scaffold coating may occur directly through interaction between molecules and surface by weak chemical bonding. Alternatively, indirectly connected after surface activation and the interposition of chemical linkers or of selective binding molecules to covalently binding molecules (Figure 3).

Biomaterials direct coating: The possibility to coat biomaterials without introducing any surface functionalization has been debated for a long time. The direct adsorption of molecules to biomaterials, otherwise called physical adsorption, occurs thanks to the interaction of protein amino acid lateral chains with the material through weak chemical bonding, i.e., hydrogen bonding and Van der Waals interactions [118,119,120,121].

A recent study by Rajabi et al. analyzed the effects of a direct laminin coating directly adsorbed onto electrospun silk fibroin/poly (-ethylene oxide) (SF/PEO) nanofibrous scaffolds on Schwann cells (SCs) proliferation and morphology. After 5 days of culture, the number of cells was significantly increased on laminin-enriched SF/PEO substrate. Furthermore, up to 5 days, cells seeded on the test scaffolds showed a spindle-like shape and extensions typical of SCs. Together, these results indicate that the coated scaffolds were more favorable for cell adhesion, growth, proliferation and for the maintenance of SCs phenotypic shape [122].

Interestingly, Noh et al. compared the osteogenic potential against human umbilical cord blood-derived mesenchymal stem cells (hUCB-MSCs) of a cell-derived ECM or of a fibronectin coating for a PLGA/PLA mesh scaffold. Noteworthy, hUCB-MSCs proliferation was enhanced both after 2 and 5 days from seeding and the expression level of osteogenic markers, i.e., bone sialo protein, collagen type 1 and alkaline phosphates, was up-regulated on ECM-derived coating, indicating a better response of cells in the presence of abundant and different cell-binding motifs [123].

The direct coating of polyethylene glycol hydrogels with RGD domain was investigated by Kudva et al. against human periosteum-derived cells. Four weeks after seeding the spreading of cells in 3D cultures was significantly ameliorated in the presence of the RGD domain, while in control group cells appeared round and without evident philopodia to adhere to the substrate. These data indicate that materials enrichment with adhesive binding motifs provide docking points for cell adhesion to trigger scaffold colonization [124].

Functionalization of material surface: It has been shown that proteins are capable of preferentially binding specific chemical groups. For instance, fibrinogen binds methyl (–CH_3_) enriched surface, but not carboxyl (–COOH) enriched ones, whereas the introduction of a hydroxyl (–OH) groups seems to enhance surface affinity for albumin over fibrinogen [125,126,127]. Thus, the presence of functionalities on scaffold surfaces may allow the control of protein adsorption. Additionally, some authors retain that the surface stability achieved through a chemical modification is much higher than that obtained by adsorption of molecules at the interface [118,128,129].

The most common way to chemically activate polymer surfaces is photochemistry. This set of techniques involves the irradiation of the surface with high-energy sources, i.e., UV, x or γ-rays, which break polymer chemical bond generating functionalities and free-radicals, which eventually lead to the propagation of the reaction [118]. Exploiting UV-light, Ma et al. were able to introduce carboxyil groups onto 3D PLLA scaffolds and to enriched them with Collagen type I from bovine tendons for cartilage TE. Subsequently, they cultured primary chondrocytes isolated from rabbit ears in the presence or in the absence of collagen coating and showed that cell proliferation was up-regulated on PLLA-Collagen scaffolds up to 6 days. Furthermore, they obtained a desirable way of adhesion and morphology for chondrocytes, since cells were more uniformly distributed and spread in the presence of collagen [19].

Alternatively, high mobile protons, which are typical of aliphatic polyesters, may be exploited to trigger hydrolyisis or aminolysis reactions [130]. To this purpose, Sadeghi et al. have recently described the possibility of modifying PLGA scaffolds with collagen, for skin TE, by testing the capacity of collagen coating to ameliorate the proliferation of human immortalized keratinocytes (HaCaT) and of human dermal fibroblasts (HDF) up to 14 days. Interestingly, the differences between the groups were significant for HaCaT cells but not for HDS, indicating that the origin of the coating has specific responses depending on the site of the application [131].

Plasma treatment is another technique to modify the surface properties of polymers. Plasma is obtained when gases are excited by specific electromagnetic frequencies and small molecules with different energies are created and cause a series of chemical changes of the substrate [118,132]. Li et al. employed the oxygen plasma to activate PLLA substrate and to enrich them with organosilanes as docking point for gelatin functionalization. Human umbilical vein endothelial cells (HUVEC) proliferation and focal adhesion expression were significantly enhances in the presence of gelatin, and were consistent with the efficiency of gelatin immobilization that depends on the organosilane used for the functionalization [133].

Grafting of selective binding molecules: Issues regarding the above cited methods are mainly due to the lack of protein mobility and to the potential immunogenicity of proteins, often isolated from other organisms [118].

To overcome these challenges, our group investigated for the first time the possibility of decorating biomaterial surfaces with selective binding molecules, able to reclaim and retain functional autologous target proteins from the surrounding milieu. Particularly, we exploited aptamers selected against fibronectin by immobilizing them on the surface of a hyaluronic acid-based hydrogel and of a chitosan membrane. We obtained selective binding materials and in both the cases we observed great fibronectin adsorption from culturing medium and an enhanced proliferation, adhesion and migration of osteoblastic cells up to 7 days of culture. Interestingly, cell response was proportional to the amount of aptamers used for the functionalization, suggesting this novel approach as a viable alternative to tailor the surface bioactivity of a scaffold for TE applications [134,135].

#### 3.4.2. Control of Cell Fate and Function

Protein immobilization on biomaterials is important to promote cell adhesion and thus scaffold colonization. However, once cells have colonized the scaffold, opportune biochemical pathways should be triggered to induce cells to accomplish a proper tissue regeneration. In this arena, cell commitment is particularly relevant and it could be controlled by enriching scaffolds with target cues.

This section will now discuss methods that have been developed to enrich scaffolds with therapeutic molecules and signals, which once delivered in the damaged site may monitor and boost regenerative processes.

**Figure 4:**
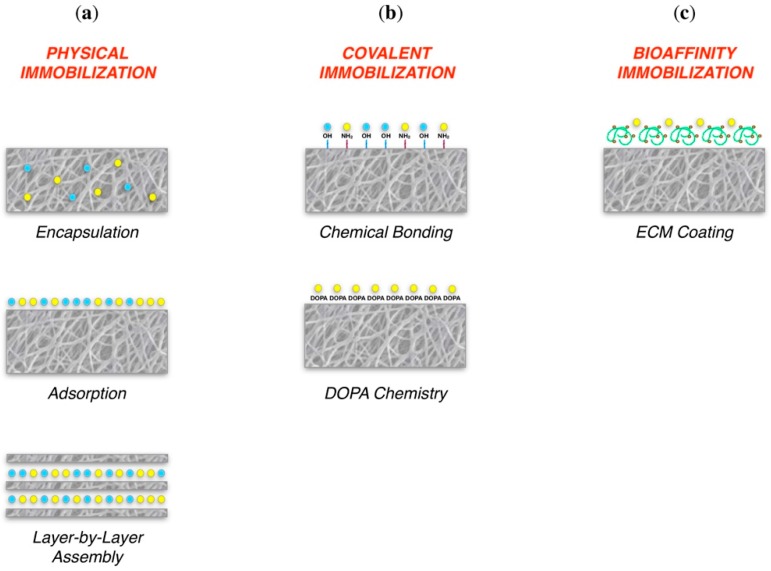
Control of cell fate and function through bioactive molecules immobilization. (**a**) Bioactive molecules may be physically immobilized on the scaffold, by encapsulation, simply adsorption or through LbL assembly; (**b**) Scaffold surface may be activated in order to covalently bind bioactive molecules; (**c**) Scaffold direct coating with ECM molecules may be exploited to bind bioactive molecules by affinity.

Among the molecules that may affect cells, growth factors (GFs) are of relevant importance. GFs are proteins secreted by committed cells, which have the ability to control cell proliferation, migration and differentiation by binding specific transmembrane receptors [136]. Numerous GFs have shown great therapeutic potential in preclinical models, including vascular endothelial growth factors (VEGFs), fibroblast growth factors (FGFs), insulin-like growth factors (IGFs), transforming growth factor beta (TGF-β), platelet-derived growth factors (PDGFs) and bone morphogenetic proteins (BMPs) [137,138,139,140]. However, do to their short half-life and consequent rapid deactivation, to date only the BMP-2 and the BMP-7 have been approved from the FDA for lumbar spine fusion and bone fracture treatment. A sophisticated alternative to the use of bare GFs in tissue regeneration may thus be their association with TE scaffolds as bare molecules (direct delivery) or combined to nanocarriers for indirect delivery, i.e., nanoparticles, nano-capsules or liposomes. Three different strategies have been until now studied for biomaterial presentation of GFs in TE: physical, covalent and bio-affinity immobilization (Figure 4) [141].

Physical immobilization: Physical immobilization techniques consists in GF retention inside scaffold structure without the formation of any chemical bond [141].

GFs encapsulation by their mixing into the scaffold before matrix gelation is the simplest method of physical immobilization and a great amount of works in the literature report this method. To this purpose, Cai et al. have recently shown the possibility to blend BMP-2 into nanometer hydroxyapatite collagen scaffolds. They detected a cumulative release of BMP-2 up to 19 days, significantly faster in the first few days. Furthermore, in the presence of BMP-2 loaded scaffold they observed an enhanced activity of alkaline phosphatase in bone marrow stem cells (BMSCs) from day 4 till day 10, indicating a pivotal role played by BMP-2 grafting in osteo-induction [142]. At this level, it could be of interest the introduction of further external stimuli that regulate scaffold biodegradation and thus bioactive cues release. This could be useful both to further control cell function and to trigger cell fate at the most appropriate moment.

Alternatively, GFs or therapeutic molecules may be simply absorbed on a matrix surface. For example, in our previous work, we showed the possibility to adsorb stanozolol on a commercially available bone substitutes and to enhance bone regeneration in a critical size defect in rat calvaria [143]. On the other hand, Wei et al. compared the possibility to mix recombinant BMP-7 (rhBMP-7) with PLGA nanospheres (NSs) and to encapsulate them into a PLLA scaffold for bone TE with the possibility of simply adsorbed rhBMP-7 on PLLA. Ectopic bone formation was clearly detectable in a subcutaneous pocket model in rat after 6 weeks of healing when rhBMP-7 was delivered with NS, while control scaffold and passive rhBMP-7 adsorbed scaffold resulted in failure of bone induction [144].

More recently, layer by layer self-assembly (LbL) strategy has been proposed as a novel method of physical immobilization. It consists in the structuration of a multilayered scaffold from the bottom to the top taking advantages from the spontaneous interaction of different charged materials [145,146]. The first LbL membrane capable of releasing BMP-2 was described in 2011 by Macdonald et al., which exploited the combination of the poly(β-aminoester), a synthetic cationic polymer, and of the cationic chondroitin sulfates. When soaked in cell culturing medium, the LbL film released the 80% of BMP-2 loaded within 2 days inducing early ALP activation in MC3T3-E1 cells (day 6), as well as enhanced calcium deposition and mineralization after 28 days. Furthermore, when implanted in an intramuscular site BMP-2 enriched LbL membranes induced bone formation and calcium deposits visible with the micro-computerized tomography analysis (µCT) from 4 to 9 weeks after implantation [147]. 

Covalent immobilization: Issues connected to the above described methods, i.e., initial burst release of immobilized molecules, may be overcome by the introduction of GFs chemical bonding to the biomaterials exploiting the reactivity of protein lateral chains functional groups. For more, this type of conjunction lead to the control of GFs desorption rate by enzymatic or hydrolytic cleavage [141]. On this way, a common approach is the creation of a polydopamine (DOPA) film on scaffold surface, by the mimicry of mussel way of adhesion to a variety of substrates [148]. DOPA chemistry has been exploited by Lee et al. to attach recombinant BMP-2 (rhBMP-2) on a 3D-printed PCL scaffold. The release of rhBMP-2 was monitored for 28 days and revealed a gradually linear release of the molecule. Furthermore, when used as cell culturing substrate the rhBMP-2 grafted scaffold did not affected cell viability, but severely influenced MC3T3-E1 cells alkaline phosphatase activity, calcium deposition and osteocalcin, bone sialoprotein and collagen type I expression, indicating a potential osteoinductive activity [149].

Bioaffinity immobilization: It should be stated that ECM act as a reservoir of GFs, which are able to bind multiple molecules with high affinity. Therefore, biomaterials could be decorated with motifs that are natural component of ECM, i.e., heparin and adhesive proteins such as fibronectin or vitronectin [141].

Tellado et al. have recently projected a biphasic silk fibroin scaffold for tendon/ligament TE functionalized with heparin for transforming growth factor β2 (TGF-β2) and growth/differentiation factor 5 (GDF5) delivery. Interestingly, heparin decoration increased the amount of TGF-β2 and GDF5 retention. Moreover, the combined delivery of these two factors promoted the chondrogenic commitment of adipose-derived mesenchymal stem cells (AdMSCs), which after 14 days showed higher expression of Sox9 and collagen type II (COL2) cartilage markers [150]. Instead, the adhesive properties of fibronectin were exploited to promote the delivery of BMP-2 from a hyaluronic acid hydrogel in an ectopic bone defect model in rat by Kisiel et al. In this case, the authors observed a 2-fold higher formation of ectopic bone and of collagen expression when BMP-2 was delivered in association with fibronectin rather than bare adsorbed on the hyaluronic acid material [151].

The scaffold requirements we have largely described in the present work are summarized in Table 1.

### 3.5. Through a Functionally Graded Scaffold

The organization that tissues, organs, apparatus and organisms have in the bulk nature is largely governed by their function, and most often it is not homogenous, but strictly arranged to combine different cells and tissues [152]. This structured organization is well-known as a functional gradient and thus during regenerative procedures, a successful TE engineered scaffold should be functionally graded (FGS) [153].

Gradients may be discrete or continuous, and may ideally be applied to mechanical, porosity and bioactivity scaffold requirements through the employment of RP techniques. For example, the articular cartilage is organized in three zones to accommodate chondrocytes according to tissue biomechanical functions. As such, the fulfillment of a scaffold with collagen fibers oriented according to native cartilage may help a faster and better regeneration of this tissue [154]. Similarly, human bones are a clear example of functional graded porosity with cortical outer layer solid and dense, while a spongy inner compound [152]. Still, it could be extremely useful to create molecular gradients to obtain a driving force that guides cell migration and differentiation [35]. These are only few of the many examples of functional gradation we may report, and it is important to considered that although the view of realizing a FGS construct is still pioneering, the way has to be that. Indeed, only matching all these perspectives it will be really possible to provide a favorable environment for cell growth, proliferation and real tissue regeneration.

## 4. A Case Study on Chitosan

Among the biological macromolecules, polysaccharides are an important class of polymers found in living organisms with structural and storage-related properties [155]. Chemically, polysaccharides are polymeric carbohydrates (sugars) formed as the result of monosaccharides polymerization, which occurs after the formation of a glicosidic bond between the hemiketal group of a saccharide and the hydroxyl group of another one.

**Figure 5:**
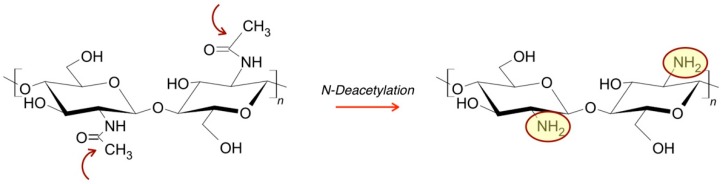
Chemical structure of chitin and of chitosan after chitin *N*-Deacetylation.

Chitin is one of the most naturally abundant polysaccharides. It is the main component of crustacean exoskeleton and of fungi/yeast/green algae cell walls, and from its partial deacetylation we can easily derive chitosan, which has been known to be a suitable candidate for TE scaffold design [156,157,158,159]. Thus, chitosan is a linear and semi-crystalline copolymer made of d-glucosamine [(1-4)-2-amido-2-deoxy-β-d-glucan] and of *N*-acetyl d-glucosamine [(1-4)-2-acetamido-2-deoxy-β-d-glucan], precisely derived from chemical hydrolysis under sever alkaline conditions or from enzymatic hydrolysis mediated by chitin deacetylase (Figure 5) [157].

Depending on the chitin source and on the chitosan preparation procedure, the degree of deacetylation (DD-d-glucosamine residues/total residues composing the chain) may range from 30% to 95%. From DD in turn depend chitosan molecular weight (300–1000 kDa) that is inversely proportional to DD, its crystallinity, which on the contrary is directly correlated to DD and chitosan stability to spontaneous degradation, which is mainly performed by lysozyme and that is delayed in high DD chitosan [159]. Furthermore, in contrast with chitin, the presence of amino groups confers to chitosan the capacity to be easily protonated, thus making chitosan the only naturally-derived polysaccharide positively charged [160]. All together, these properties make chitosan a suitable biocompatible biomaterial for TE scaffold design.

Protonated groups provide solubility in acidic aqueous solution and allow chitosan adaptability under a wide spectrum of conditions, thus endorsing it with a unique structural versatility [161]. Starting from chitosan solution, hydrogels, porous (sponges) or fibrous scaffolds have been prepared for different applications [157].

Chitosan is capable to jellify all by itself and physical reversible hydrogels have been realized by controlling specific parameters (e.g., pH, temperature, etc.) to create interactions between polymer chains [157,162,163]. Alternatively, positively charged d-glucosamine residues may interact with negatively charged molecules (e.g., sulfates, phosphates, etc.) to form hydrogels [157]. The main advantage of this type of gel is that their gelation is easy and may occur upon hydrogel injection offering the possibility to perfectly shape the scaffold to the tissue defect. On the other hand, both the amino and the hydroxyl groups of chitosan lateral chains may be used to form un-reversible hydrogels via covalent bonding (i.e., amide, ester or Schiff base formation) of chemical cross-linkers, thus forming more stable hydrogels [44,164]. Chemical cross-linking agents such as glyoxal or glutaraldehyde have been for a long used, but their high toxicity has led to the introduction of other natural components (i.e., genipin) [165].

Porous chitosan sponges could be also obtained, generally by freeze-drying and by gas foaming, or fibrous structures through solvent casting, leaching techniques, electrospinning and bottom-up approaches (e.g., 3D-bioprinting) [163,166,167,168,169].

As we stated above in this work, the manufacturing technique allow to control scaffold porosity, which is important to ensure proper nutrient, oxygen and waste diffusion to and from cells. Focusing on chitosan, pores play also an important role in defining suitable mechanical properties. In a detailed work, Madihally and Matthew described the correlation between porosity and rheological parameters in chitosan models [161]. They showed that hydrated porous chitosan membranes had a greatly reduced elastic module tensile strength if compared to non-porous ones (0.1–0.5 MPa vs. 5–7 MPa), while maximum strain ranged from 30–40% for non-porous membranes to 30–110% (depending on diameter and orientation) for the porous. Furthermore, in another work by Chen and Hwa, the effect of chitosan molecular weight on mechanical properties was investigate [170]. Findings revealed that both the tensile strength and the elastic modulus were directly proportional to chitosan molecular weight. As a consequence, chitosan mechanical properties indirectly depend also on DD and crystallinity, while directly from the biodegradation kinetics, as more fast the degradation occur, as more fast the chitosan loose its mechanical stability [159].

The cationic nature of chitosan could be further exploited for the preparation of multilayered films, using LbL technique or for chitosan functionalization with anionic glycosaminoglycans and proteoglycans widely present in natural ECMs. As we stated before (see Section 3.4.2), both these methods could be useful for the reception of suitable molecules in guiding cell fate and function [171,172,173].

One of the important features of chitosan is the presence of three distinct reactive functional groups along its molecular chain. Indeed, the two hydroxyl groups in C3 and C6 positions and the amine group in C2 position make chitosan flexible for chemical modifications and for the amelioration of its bioactivity through proteins and therapeutic molecules grafting [174].

Fascinatingly, it has been recently described the possibility to introduce suitable chemical groups for chitosan functionalization by enzymatic modifications [175]. Enzymatic modifications are gaining remarkable attention to modify the structure of chitosan because they are selective, specific and they do not require harsh conditions or reactive and toxic compounds. Such procedures have been already successfully used to graft proteins and other suitable molecules on chitosan surface [176,177].

### 4.1. Chitosan in Tissue Engineering

As we have widely stated above, the selection of an appropriate material for biocompatible scaffold design is a critical step in TE. To this purpose, thanks to its beneficial properties, chitosan has been extensively studied as raw materials for scaffold fabrication.

To the best of our knowledge, more than 2000 works have been published in the last 20 years with an increasing exponential trend from 1997 to the end of 2017. Among these works, an important cut is covered by works on bone TE (43.60%), followed by skin (16.80%) and cartilage (14.50%) TE.

#### 4.1.1. Bone

Unlike other tissues, bone has a great capacity to regenerate. Nevertheless, in some situations, such as when important traumas occur or after bone tumor resections, massive bone defects may be created, which need a clinical intervention. Furthermore, possible insufficient blood supply, the presence of infections or of systemic disease (e.g., diabetes) can negatively affects bone healing, resulting in an ineffective regeneration also for contained defects [178,179].

In these cases, the selection of an opportune bone graft has been for a long the gold standard therapeutic approach, including the use of autografts, allografts and xenografts. However, issues related to the used of these substitutes, such as the requirements of a second donor site or the prolonged operative times in the case of autografts and potential immune response in the case of allo- and xenografts, have prompted efforts in bone TE by developing alloplastic and synthetic scaffolding materials [178,179,180].

As for other tissues, the ideal scaffold for bone TE should fulfill specific requirements and among the most important there are (i) osteoinduction, otherwise the capability to recruit and stimulate bone-forming cells, (ii) osteoconduction, which is the ability to support cell adhesion, (iii) osteogenesis that consists in the formation of new bone and (iv) mechanical stability [178,181,182].

In this regard, it has been shown that chitosan similarities with natural biological tissues is useful to trigger the proliferation and differentiation of mesenchymal cells in osteoprogenitors [183]. Thus, 3D chitosan-based scaffolds for bone applications have been prepared by solvent casting, gas foaming, freeze-drying and RP prototyping, finding good outcomes both in vitro and in vivo applications [184,185,186].

One of the latest researches from the literature includes an impressive work by Gan et al, which prepared a chitosan scaffold whose biocompatibility was implemented at further levels [187]. In particular, biphasic calcium phosphate was used to enhance mechanical properties, RGD-binding motifs to promote scaffold colonization by cells and BMP-2 loaded bovine serum albumin NPs were used to confer osteoinductive properties to the construct. The scaffold was prepared by freeze-drying and exhibited optimal characteristics for bone TE: a pore size ranging from 10 to 100 µm and a compressive strength between 150 and 420 GPa. Furthermore, the amelioration of scaffold bioactivity by RGD immobilization via carbodiimide crosslinking chemistry and by BMP-2 loaded BSA NPs by dispersion in chitosan solution, contribute to trigger mesenchymal stem cells proliferation and differentiation in vitro and bone regeneration in a femur defect in vivo. Noteworthy, Masson’s trichromatic staining revealed the presence of organized collagen fibers and of enhanced bone regeneration around the defect region after 12 weeks in chitosan-RGD-BMP-2 scaffold, while no bone, but fibrous tissue was detected in a chitosan-RGD scaffold and immature bone in chitosan-BMP-2 scaffold.

#### 4.1.2. Skin

As a consequence of skin lesions the complete regeneration of a functional epithelium is more than ever important to ensure its role of barrier, pigmentory defense against UV irradiation, thermoregulation, as well as mechanical and aesthetic functions [188].

Cutaneous wound healing requires a well-orchestrated coordination between biological and molecular events which start with tissue hemostasis to conclude with epidermis new formation [189,190]. However, while small defects can heal spontaneously, large and important lesions (i.e., burns) need a surgical resection of the necrotic tissue and consequent positioning of a skin grafts. Furthermore, an impairment in skin healing processes progression, normally due to presence of systemic disease (i.e., diabetes), may induce chronic wounds, which lead to an impairment of local cytokines production and to a consequent reduction in angiogenesis and wound vascularization [191].

As for bone, many skin substitutes have been used in the past decades to promote wound healing, but for issues connected to antigen susceptibility and limitation of donor sites, synthetic approaches have been developed often in combination with therapeutic cytokines and GFs [13].

Chitosan, which among its properties is capable to trigger hemostasis and platelet activation, is one of the eligible materials for the preparation of wound dressing substances and numerous materials have been already approved for commercial trade (e.g., Chitoseal^®^—Abbott, Syvec Patch^®^—Marine Polymer Technologies, Chitopoly^®^—Fuji Spinning) [192]. However, even though these products have been shown to promote wound healing, they do not overcome the issue of wound chronicization and in this sense, research efforts to find optimal products for diminished wound healing capacities are still a major goal in skin TE. To this purpose, in a recent work, Wu et al. have successfully applied a chitosan/silk fibroin patch co-seeded with adipose-derived stem cells (ADSCs) as reconstructive support in diabetic wound healing. They showed a great capacity of the patch to enhance wound healing rate, with statistically significant differences up to 21 days, as well as a promotion of angiogenesis with blood vessels growing perpendicular to the wound [193].

#### 4.1.3. Cartilage

Joint degeneration is progressive with aging and its treatment represents a major challenge for clinicians, because articular cartilage self-regeneration is highly limited due to a lack of vascularization and to a limited proliferative capacity of chondrocytes [194,195].

In the past decades, the transplantation of autologous chondrocytes seemed to be a promising procedure, but scarce cell population together with their restricted ability to expand, led in any case to numerous difficulties in repairing large critical defects [196,197]. Thus, the most common option to restore joint surface is nowadays the invasive surgical activation of subchondral bone healing through its injury by drilling, micro fractures realization, arthroplasty or electrical/laser stimulation, to promote the release and the activation of resident mesenchymal stem cells (MSCs) [198]. However, MSCs are an attractive cell source for TE, which can be easily harvested in large amounts from a variety of sources, including adipose tissue, umbilical cord blood, placenta and bone marrow. As such, to overcome drawbacks related to methods for cartilage repair, the way of isolating MSCs from other autologous sources and to seed them on chondrogenic scaffolds has been deeply considered.

The structural similarity of chitosan to natural components of cartilage ECMs, including collagen type II and GAGs, which have been known to play a central role in chondrogenesis orchestration as well as in guiding chondrocytes fate, makes it a suitable scaffolding material for cartilage repair [199,200,201].

Among the latest advances in the literature on this topic, Deng et al. prepared a chitosan and silk fibroin based scaffold to culture and differentiate primary bone marrow MSCs, prior to construct implant in articular cartilage defects in rabbits [202]. Up to 12 weeks of healing they showed that a chitosan/silk fibroin scaffold may serve as a carrier for MScs to repair cartilage defects and may be further exploited in TE approaches.

#### 4.1.4. Cornea

The cornea is an extremely organized multi-layered structure that allows light to enter in the eye and that constitutes a vital component of the vision system [203]. Corneal blindness is the first cause of vision loss and it is estimated that 4.9 million people worldwide suffer from bilateral blindness, while 5–23 million suffer from unilateral blindness [204]. To date, the standard treatment for restoring vision after cornea injury is its transplantation from cadaveric donors (keratoplasty), but in fact there is a growing need for therapeutic options, due to the high risk of graft rejection and to the chronic lack of donors [205]. As such, TE approaches of full or partial portions of the cornea have represent a viable alternative in the last ten years, with particular regard to constructs supporting corneal epithelium, stroma and endothelium regeneration. More often, these approaches have considered chitosan as a suitable biomaterial for scaffold fabrication, which is already a widely used polysaccharide in ophthalmic applications.

The epithelium is the most anterior portion of the cornea, as the most susceptible to accidental damage. In physiological conditions, damages are normally repaired by limbal epithelial stem cells, but in case of their loss (i.e., limbal stem cell deficiency—LSCD), neovascularization and conjunctiva outgrowth occur causing epithelium opacization and vision loss [206]. As TE approaches, the latest work from the literature by Xu et al. proposed the preparation of a carboxymethyl chitosan/gelatin/hyaluronic acid-blended membrane to graft a new-forming epithelium from primary rabbit corneal epithelial cell in alkali-induced corneal damages [207]. This study revealed that the membrane was transparent and suitable to support cell growth and phenotype. Furthermore, the combination of the membrane with cells was a successful achievement for corneal epithelium wound healing, showing quite a complete restoration of the initial structure.

Under the corneal epithelium, the stroma constitutes the 80–90% of cornea thickness. This structure is organized in layers of well aligned collagen bundles and keratocytes, which confer to the epithelium the proper hydration and to the entire cornea proper mechanic and transparency [208]. In case of stroma lesions, the reproduction of such complex collagen architecture is a demanding challenge for TE. To this purpose, in a successful work Guan et al. were able to apply a chitosan/silk fibroin scaffold for corneal stroma engineering in rabbits and to obtain a reconstructed lamellar structure comparable to that of native tissue: transparent and expressing vimentin in keratocytes [209].

Eventually, the corneal endothelium is the deeper portion of the cornea. It is a monolayer of cells arrested in G_1_ phase of the cell cycle and it is responsible for fluid exchange with the stroma. Due to the incapacity of endothelial cells to proliferate, normal aging causes a gradual decrease in cell number with consequent thickness of the endothelium until, in some cases, blindness [210]. As for stroma, the engineering of this portion is nowadays demanding, and few works have been proposed to fulfil with an effective endothelium. Among others, a work by Liang et al. showed the possibility to develop a chitosan-based membrane able to support primary endothelial cells from rabbit growth and morphology [211].

## 5. Conclusions and Future Perspectives

The choice of suitable biomaterial for scaffold fabrication represents a key point in TE. Through the integration of multiple discipline approaches, tremendous developments have been done in the last decade for the realization of scaffolds both in vitro and in vivo.

In this review, we highlighted the requirements in terms of biodegradability, mechanical properties, porosity and bioactivity that a biomaterial scaffold should have to fulfill functional tissue regeneration. In particular, we emphasized how these aspects should be tailored in order to reflect the physiological needs of the regenerating tissue and of its surrounding milieu. In addition, the chitosan has been reported as a suitable and versatile biomaterial in order to provide the concrete evidence of the possibility to mold the same material for tissues with far anatomies and physiologies. Based on these advances, we expect that the future directions of TE will continue to combine these aspects in order to obtain functional graded biological substitutes to boost even more tissue regeneration.

## Figures and Tables

**Table 1:** Scaffold requirement to design effective scaffolds for tissue engineering.

Scaffold Requirement	Biological Significance	How to Control Scaffold Requirement	Example	Reference
**Biodegradability**	Once placed, the scaffold should be reabsorbed in order to: (i). leave space to the new regenerating tissue; (ii). avoid undesired effects (i.e., internal pores obstruction with consequent tissue necrosis).	Synthetic polymers	Bone TE Cartilage TE Periodontal TE Muscle TE Skin TE Neural TE	Cai et al., 2018 [141] Ma et al., 2005 [19] Galli et al., 2016 [133] Zahari et al., 2017 [57] Sadeghi et al., 2016 [130] Rajabi et al., 2018 [121]
		Natural polymers	Bone TE Ligament TE Periodontal TE Skin TE Neural TE	Kudva et al., 2018 [123] Font Tellado et al., 2018 [149] Parisi et al., 2017 [134] Shefa et al., 2017 [58] Li et al., 2017 [41]
**Mechanical properties**	The scaffold should be mechanistically similar to the tissue to regenerate in order to: (i). maintain the integrity of the defect until complete regeneration; (ii). possess fatigue property when undergoes to cyclic loading.	Porous scaffolds	Neural TE	Li et al., 2017 [41]
		Hydrogels	Cartilage TE Neural TE	Zhang et al., 2018 [51] Chedly et al., 2018 [52]
		Fibrous scaffolds	Bone TE Cartilage TE Ligament TE Muscle TE Skin TE	Khorouschi et al., 2018 [54] Chen et al., 2016 [55] Pauly et al., 2016 [56] Zahari et al., 2017 [57] Shefa et al., 2017 [58]
**Porosity**	The scaffold should be porous in order to: (i). allow the transport of nutrients and waste to and by cells, respectively; (ii). allow cell ingrowth.	Top-down approaches	Cartilage TE Skin TE Bladder TE Cardiac TE	Wimpenny et al., 2012 [69] Zonari et al., 2014 [73] Korossis et al., 2009 [70] Baheriraei et al., 2015 [76]
		Bottom-up approaches	Cartilage TE	Yingying et al., 2017 [96]
**Bioactivity**	The scaffold should be bioactive in order to: (i). support cell adhesion and scaffold colonization; (ii). support cell fate and function.	Direct coating	Bone TE Neural TE	Noh et al., 2016 [122] Kudva et al., 2018 [123] Rajabi et al., 2018 [121]
		Surface functionalization	Cartilage TE Skin TE	Ma et al., 2005 [19] Sadeghi et al., 2016 [130]
		Selective binding molecules	Periodontal TE	Galli et al., 2016 [133] Parisi et al., 2017 [134]
		Physical immobilization: (i). Encapsulation (ii). Adsorption (iii). Layer-by-layer assembly	Bone TE	Cai et al., 2018 [141] Ghiacci et al., 2017 [142] Wei et al., 2007 [143] Macdonald et al., 2011 [147]
		Covalent immobilization: (i). Chemical bonding; (ii). DOPA chemistry.	Bone TE	Lee et al., 2016 [149]
		Bioaffinity immobilization	Bone TE Ligament TE	Kisiel et al., 2013 [151] Font Tellado et al., 2018 [150]
